# Supplementary material for: Terahertz Spectroscopic Identification of Roast Degree and Variety of Coffee Beans
Source: Foods. 2024 Jan 24;13(3):389. doi: 10.3390/foods13030389 (PMC10855191; doi:10.3390/foods13030389)
Supplement: Supplementary file 1 [file foods-13-00389-s001.zip › foods-2758130-supplementary.pdf]

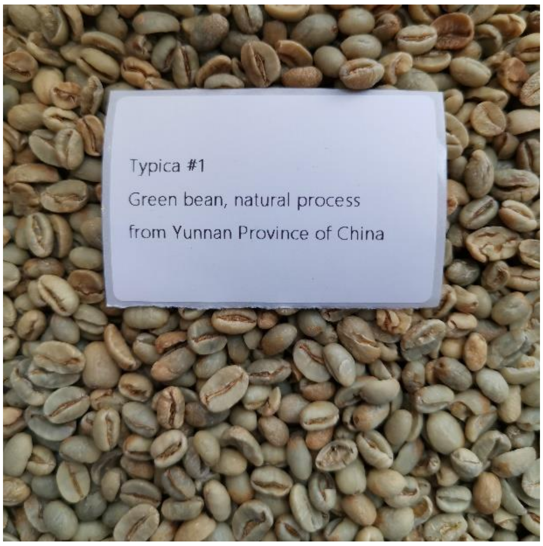
Typica #1
Green bean, natural process
from Yunnan Province of China

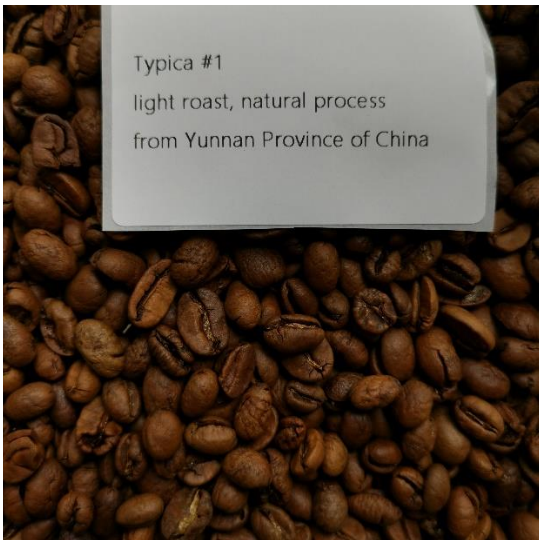
Typica #1
light roast, natural process
from Yunnan Province of China

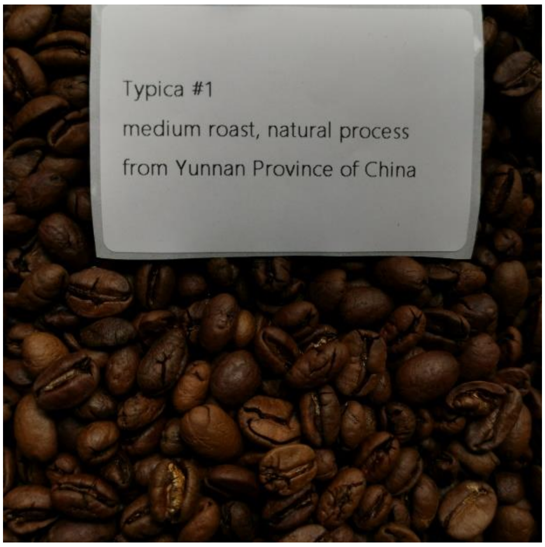
Typica #1
medium roast, natural process
from Yunnan Province of China

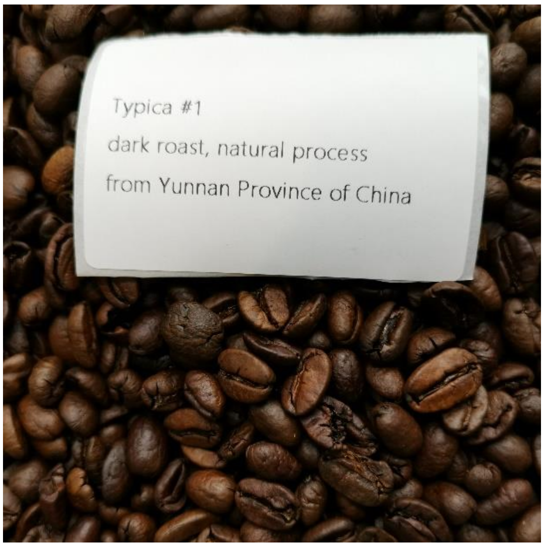
Typica #1
dark roast, natural process
from Yunnan Province of China

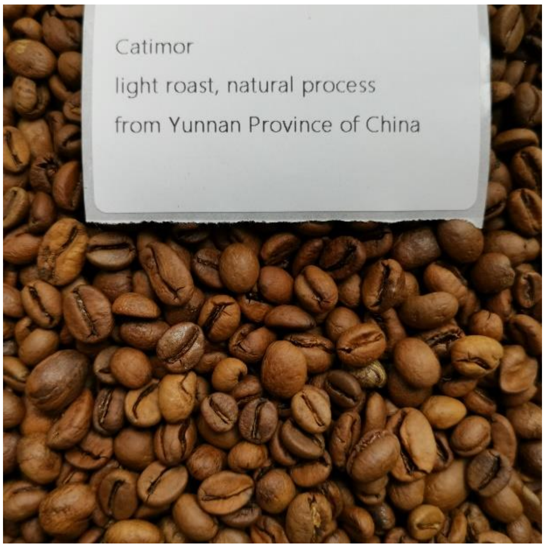
Catimor
light roast, natural process
from Yunnan Province of China

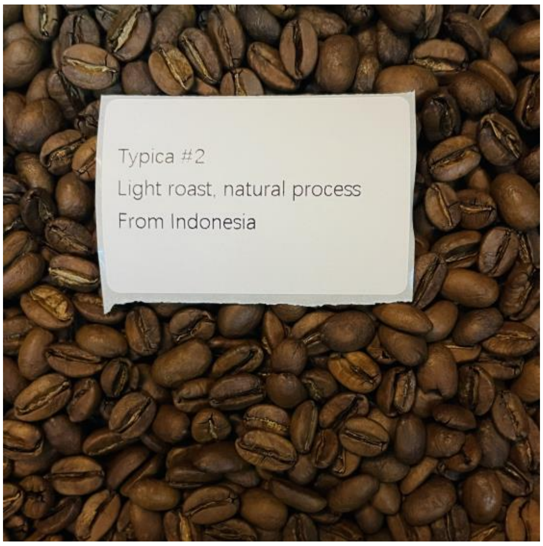
Typica #2
Light roast, natural process
From Indonesia
